# Machine learning and deep learning in diabetology: revolutionizing diabetes care

**DOI:** 10.3389/fcdhc.2025.1547689

**Published:** 2025-05-27

**Authors:** Salvatore Corrao, Miodrag Janić, Viviana Maggio, Manfredi Rizzo

**Affiliations:** ^1^ Department of Clinical Medicine, Internal Medicine Unit, National Relevance and High Specialization Hospital Trust Azienda di Rilievo Nazionale ed Alta Specializzazione (ARNAS) Civico Di Cristina Benfratelli, Palermo, Italy; ^2^ School of Medicine, Promise Department of Health Promotion Sciences Maternal and Infantile Care, Internal Medicine and Medical Specialties, University of Palermo, Palermo, Italy; ^3^ Department of Endocrinology, Diabetes and Metabolic Diseases, University Medical Centre Ljubljana, Ljubljana, Slovenia; ^4^ Faculty of Medicine, University of Ljubljana, Ljubljana, Slovenia; ^5^ Internal Medicine Department, Ras Al Khaimah College of Medical Sciences, Ras Al Khaimah Medical and Health Sciences University, Ras Al Khaimah, United Arab Emirates

**Keywords:** machine learning, deep learning, artificial intelligence, diabetes management, challenges

## 1 Introduction

Diabetes is a chronic disease affecting over 400 million people globally, with its prevalence expected to rise sharply in the coming decades due to ageing populations, sedentary lifestyles, and increasing obesity rates (1). It is a leading cause of serious complications, including cardiovascular disease, nephropathy, neuropathy, and retinopathy, contributing significantly to morbidity, mortality, and healthcare costs (2, 3). Traditional approaches to diabetes management often fail to address the complexity of the disease, particularly in cases involving highly variable glucose patterns or multiple comorbidities (4–6).

The emergence of artificial intelligence (AI), particularly machine learning (ML) and deep learning (DL), has introduced new possibilities in diabetology. While ML focuses on analyzing structured datasets to identify patterns and trends, DL extends this capability by processing unstructured and multimodal data, such as retinal images and continuous glucose monitoring (CGM) outputs (7, 8). These technologies create a paradigm shift in diabetes care, empowering clinicians and patients with precise, data-driven insights.

This opinion focuses on the applications, methodological differences, and critical advancements in using ML and DL in diabetes. With the exponential growth of healthcare data generated from wearable devices, CGMs, and electronic health records (EHRs), there is immense potential to enhance diabetes management. However, the complexity of this chronic disease demands more sophisticated tools than traditional methods can offer.

## 2 Differences between machine learning and deep learning

ML and DL are subfields of AI that share a common goal of enabling machines to learn from data, yet they differ fundamentally in their methodologies, complexity, and applications. ML focuses on algorithms that analyze structured data and make predictions or decisions based on these patterns. Standard techniques include logistic regression, decision trees, support vector machines (SVMs), and random forests. These models typically rely on manual feature extraction, where domain experts determine the most relevant variables for analysis (9, 10).

In contrast, DL uses artificial neural networks inspired by the human brain to process data hierarchically. It excels in analyzing unstructured data, such as images, text, and sequential data, without explicit feature engineering. Architectures like convolutional neural networks (CNNs) are particularly effective for image analysis. At the same time, recurrent neural networks (RNNs) and long short-term memory (LSTM) networks are widely applied to time-series data (11, 12). DL models, however, demand significantly larger datasets and greater computational power than traditional ML approaches, making them more resource-intensive (13).

**Figure 1:**
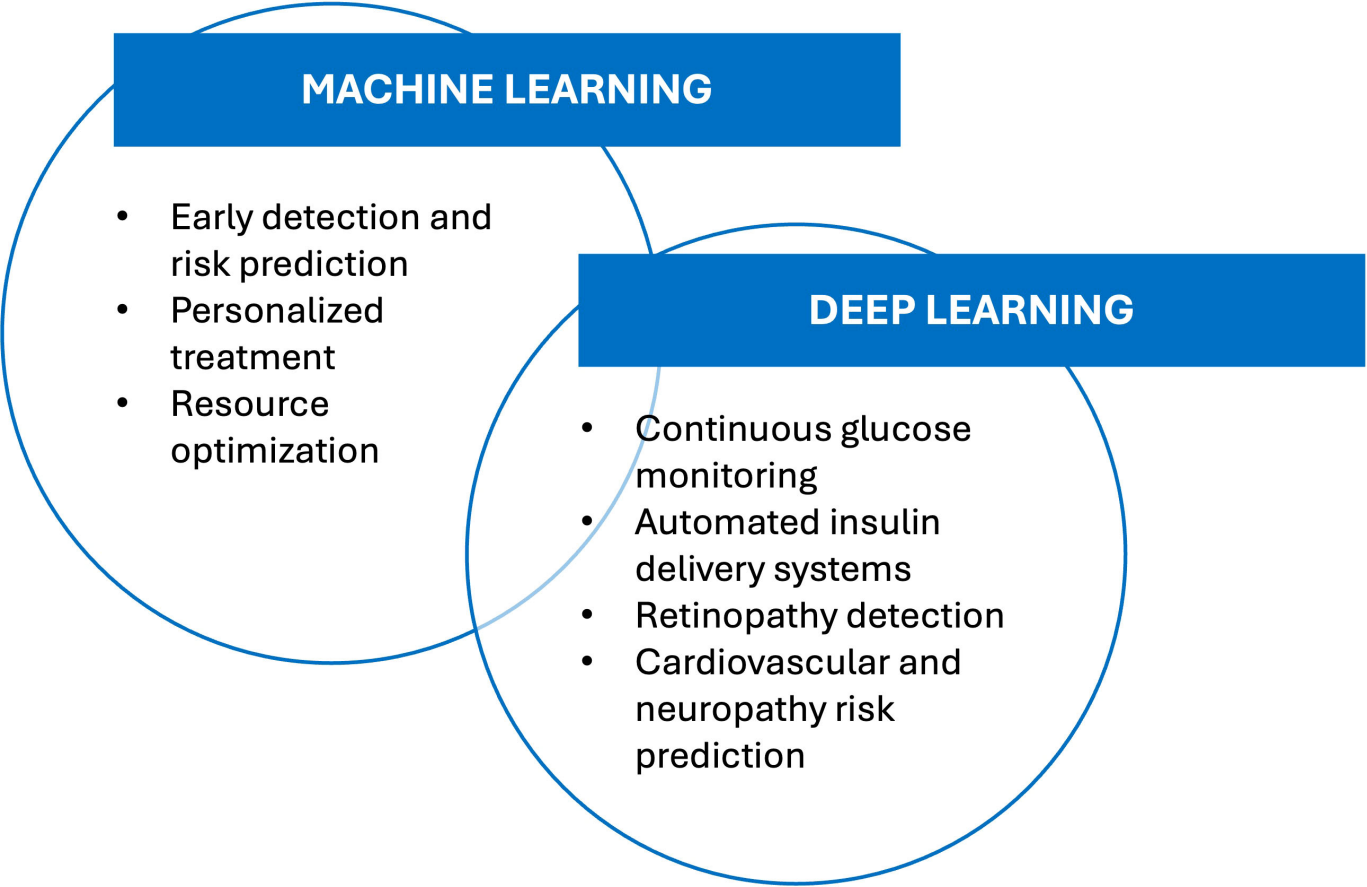
Applications of machine learning and deep learning in the practice of diabetology (see the text for more explanation).

The choice between ML and DL depends mainly on the nature of the problem, data type, and computational resources. ML is often preferred for tasks involving smaller datasets and requiring interpretability, such as predicting diabetes risk based on clinical and demographic data. DL, by contrast, is better suited for high-dimensional data analysis, such as detecting diabetic retinopathy from retinal fundus images or forecasting glucose fluctuations from CGM readings (14, 15). These differences underscore the complementary roles of ML and DL in advancing diabetes care as depicted in **Figure 1**.

## 3 Applications of machine learning in diabetology

### 3.1 Early detection and risk prediction

ML algorithms are pivotal in identifying individuals at risk of developing type 2 diabetes or its complications. Logistic regression and random forest models, for example, analyze demographic and clinical factors, such as body mass index (BMI), fasting glucose, and family history, to calculate risk scores (16, 17). Moreover, these models have been employed to stratify patients based on their likelihood of developing complications like diabetic retinopathy or nephropathy, facilitating timely medical interventions (18).

### 3.2 Personalized treatment

Personalized medicine is another transformative application of ML in diabetology. To optimize treatment regimens, algorithms analyze patient-specific data, such as glucose patterns, activity levels, and dietary intake. Ensemble learning models have successfully predicted insulin dosages tailored to individual needs, reducing the risks of hypoglycemia and hyperglycemia (19, 20).

### 3.3 Resource optimization

ML also addresses resource challenges in healthcare systems by predicting hospitalization risks and prioritizing high-risk patients for follow-up care. EHR-based predictive models enable clinicians to allocate resources effectively, ensuring timely interventions for those with poorly controlled diabetes (21).

## 4 Applications of deep learning in diabetology

### 4.1 Continuous glucose monitoring

DL has significantly enhanced CGM systems by improving the accuracy of glucose prediction models. RNNs and LSTM networks analyze sequential glucose data to forecast levels several hours ahead, enabling patients to adjust insulin or carbohydrate intake proactively (22–24).

### 4.2 Automated insulin delivery systems

DL algorithms power artificial pancreas systems by integrating CGM data with insulin pump controls. CNNs detect patterns in glucose trends, automating insulin delivery with precision. Clinical trials have shown that these systems improve glycemic control and reduce HbA1c levels (25).

### 4.3 Retinopathy detection

CNNs are widely used in analyzing retinal fundus images for the early detection of diabetic retinopathy. These models have achieved diagnostic accuracies comparable to human ophthalmologists, making them particularly valuable in resource-constrained settings (12, 26).

### 4.4 Cardiovascular and neuropathy risk prediction

DL models also help assess the risks of diabetic complications, such as cardiovascular diseases and neuropathy. Transformer-based architectures, such as BERT, combine data from wearable devices, clinical notes, and EHRs to provide comprehensive risk assessments (17).

## 5 Challenges in implementing machine learning and deep learning in diabetology

Despite their transformative potential, adopting ML and DL in clinical settings faces several challenges. Data quality and standardization remain significant barriers, as healthcare datasets often need to include more consistent values (10). Moreover, the “black box” nature of DL models limits their interpretability, making it difficult for clinicians to trust and adopt these tools (27). Ethical issues, including data privacy and biases in training datasets, further complicate their implementation (28, 29).

Emerging solutions like federated learning allow decentralized model training without compromising patient privacy. Explainable AI (XAI) initiatives are improving transparency fostering greater trust among healthcare professionals (30).

## 6 Discussion

This opinion provides actionable insights by discussing AI’s opportunities in diabetology and critically analyzing its current limitations. Researchers, clinicians, and healthcare professionals should be aware how these transformative technologies can significantly improve the lives of people with diabetes as ML and DL revolutionize diabetes management by enabling early detection, personalized treatment, and advanced monitoring.

Recent advances in artificial intelligence have extended beyond individualized care to offer strategic insights into diabetes care trends at the population level. AI tools, particularly interpretable machine learning models such as the Logic Learning Machine, have been successfully applied to identify predictors of lipid goal attainment in large outpatient cohorts with type 2 diabetes, as demonstrated by the AMD Artificial Intelligence Study Group (31). Similarly, deep learning applied to electronic health records has enabled the prognostic modelling of incident heart failure among diabetic patients, offering an early warning system for adverse cardiovascular outcomes (32). In another large-scale application, transparent ML algorithms have revealed patterns of therapeutic inertia in HbA1c trajectories, shedding light on modifiable gaps in diabetes management at the system level (33). These findings underscore the expanding scope of AI in addressing not only personalized medicine, but also healthcare planning, quality assessment, and intervention targeting on a broader scale.

The future of ML and DL in diabetology is promising. While challenges related to data quality, interpretability, and ethics persist, advances in wearable technology and real-time data integration will further enhance monitoring capabilities, overcoming these barriers. Federated learning frameworks will facilitate collaboration between institutions, while XAI will make AI tools more interpretable. Continued investment in AI infrastructure and interdisciplinary research will be critical to realize the full potential of these technologies to transform diabetes care. Integrating AI into clinical workflows will undoubtedly improve outcomes and enhance the quality of care for millions of individuals living with diabetes.
